# An Explorative Study into the Aetiology of Developmental Dysplasia of the Hip Using Targeted Urine Metabolomics

**DOI:** 10.3390/antiox12030538

**Published:** 2023-02-21

**Authors:** Amanda M. L. Rhodes, Sehrish Ali, Magdalena Minnion, Ling H. Lee, Brijil M. Joseph, Judwin Ndzo, Nicholas M. P. Clarke, Martin Feelisch, Alexander Aarvold

**Affiliations:** 1 Orthopaedic Surgery, University Hospital Southampton, Southampton SO16 6YD, UK; 2 Clinical and Experimental Sciences, Faculty of Medicine, University Hospital Southampton, Southampton SO16 6YD, UK; 3 Southampton Children’s Hospital, University Hospital Southampton, Southampton SO16 6YD, UK; 4 Department of Paediatric Orthopaedics, University of Southampton, Southampton SO16 6YD, UK

**Keywords:** reactive species, oxidative stress, hydrogen sulphide, redox processes, sulphate, skeletal development

## Abstract

Developmental dysplasia of the hip (DDH) is the most prevalent congenital musculoskeletal disorder, yet its cause remains unknown. Adequate nutrient provision and coordinated electron exchange (redox) processes are critical for foetal growth and tissue development. This novel study sought to explore specific biochemical pathways in skeletal development for potential involvement in the aetiology of DDH. Spot urine samples were collected from infants, aged 13–61 days, with and without DDH. Ion chromatography-mass spectrometry was used to quantify thiosulphate, sulphate, nitrate, and phosphate, whilst nitrite was quantified using high-performance liquid chromato-graphy. Thiobarbituric acid reactive substances (TBARS) were measured as markers of lipid peroxidation. Creatinine and osmolality were determined by a 96-well plate assay and micro-osmometer to potentially normalise values for renal function, lean body mass, and hydration status. Urine samples were analysed from 99 babies: 30 with DDH and 69 age-matched non-DDH controls. Thiosulphate, TBARS, and creatinine concentrations differed between the DDH group and the controls (*p* = 0.025, 0.015, and 0.004 respectively). Urine osmolality was significantly lower in DDH compared to the controls (*p* = 0.036), indicative of the production of a more diluted urine in DDH infants. Following adjustment for osmolality, significant differences became apparent in urinary sulphate levels in DDH (*p* = 0.035) whereas all other parameters were similar between the groups. This is the first study to assess the potential role of these inorganic anions in DDH. The higher levels of sulphate found in infants with DDH suggests either enhanced intake from milk, increased endogenous formation, or impaired renal reabsorption. This investigation demonstrates the power of urine metabolomics and highlights the importance of normalisation for hydration status to disentangle developmental disorders. Our results strongly suggest that DDH is a systemic disease associated with altered uptake, formation, or handling of sulphate. There is potential for new opportunities in the prevention or treatment of DDH via nutritional intervention.

## 1. Introduction

Developmental dysplasia of the hip (DDH) remains the most common hip abnormality in infants, with a UK incidence of 4.9 per 1000 live births [1]. As the leading cause of early onset hip osteoarthritis in young adults, it is responsible for 29% of primary hip arthroplasty in patients under 60 years of age [2]. Despite huge costs to both healthcare and patients, the aetiology of DDH remains poorly understood. Although no clear genetic cause has been established, recent evidence points to the involvement of aberrant epigenetic regulation including DNA hypermethylation affecting gene expression [3], the trigger of which is unclear. Although the anatomical characteristics of bony structures in DDH have been studied extensively, much less is known about alterations in soft tissues [4], and there is a paucity of evidence on the role of potential hormonal, nutritional, and chemical factors [5,6,7].

An important aspect of the pathogenesis of musculoskeletal disease is disruption of the physiological processes involved. This is well understood in some skeletal dysplasias such as achondroplasia, mucopolysaccharidoses, and Gaucher’s disease, with medical treatments subsequently developed. Yet for DDH, which is far more prevalent, the aetiology remains unknown. Oxidative stress/redox regulation is a rapidly evolving area of biological research. It is linked to skeletal growth and development via the sulfation of glycosaminoglycans [8] and other fundamental processes of cell regulation and developmental biology. However, this has never been investigated in relation to DDH, the most common dysplasia of the musculoskeletal system.

Several related disorders, including osteoarthritis, are associated with altered nitric oxide (NO) formation/metabolism and oxidative stress [9,10]. The fluctuating oxygen tension with concomitant variation in oxidant production in the synovial fluid results from a pathological increase in tissue metabolism, an abnormal strain on the joint, and ischaemia–reperfusion-related phenomena [9,11]. In a recent study, Altay et al. found significantly higher oxidative stress and lower antioxidant levels in participants’ serum with DDH [12]. Since reactive oxygen species (ROS) can interfere with sulphur-based cell signalling and metabolic regulation, sulphur biology is a compelling area for research.

Sulphur is an essential nutrient, necessary for many crucial physiological functions, in particular during growth and skeletal development. It occurs in a range of oxidation states, from the most reduced (hydrogen sulphide, H_2_S) to the most oxidised (sulphate) forms, with thiosulphate arising as a key intermediate of H_2_S oxidation in mitochondria. Sulphur is responsible for the maintenance of the structure and function of tissue (glycosaminoglycans), drug detoxification, phase II metabolism (conjugation reactions), and the inactivation of numerous hormones and neurotransmitters [13,14]. The main source of sulphur is from dietary intake, either in the form of inorganic sulphate or plant/animal-based protein. There is a growing body of evidence supporting the benefits of naturally occurring sulphur-containing molecules (organosulphur compounds) on skeletal health [15].

The intersection of oxygen, nitrogen, and sulphur metabolism is highlighted by the chemical interaction of short-lived ROS (mediators of ‘oxidative stress’), reactive nitrogen species (RNS; including the product of arginine oxidation, NO), and reactive sulphur species (RSS; including H_2_S formed from methionine, homocysteine, and cysteine) [16]. A rich network of chemical interactions between small volatile molecules (coined ‘gasotransmitters’, including NO and H_2_S) and their biological targets, known as the ‘reactive species interactome’ (RSI) [16] are responsible for the ability of cells, organs, and whole organisms to sense and adapt to alterations in environmental and metabolic conditions. The RSI may also be involved in the coordination/optimisation of nutrient supply and use during growth [17]. Although many of the complex molecular processes involving sulphur biochemistry are not fully understood yet, electron exchange (reduction and oxidation, i.e., redox) processes are fundamental to human physiology and stress resilience [17,18]. Like most other physiological processes, bone formation and bone metabolism (via osteoblasts and osteoclasts) are influenced and regulated by cellular redox status [19]. Both the formation and degradation of cartilage (collagen) are also tightly controlled by redox-dependent processes [20,21]. Phosphate is another essential body mineral that is known to be crucial for cell signalling, skeletal development, cellular energy provision, and bone integrity. Low levels of phosphate can result in skeletal abnormalities following delayed growth and rickets [22], which forms the rationale for its inclusion in fortified milk products to improve bone mineralisation and growth in premature infants [23].

The susceptibility to developing DDH is likely to have its origins in utero. The hip joint begins to develop in the foetal phase at week 8. This, along with high nutrient and energy demands in early pregnancy by the developing foetus, prompts maternal circulating concentrations of sulphate to increase roughly two-fold. This is achieved by increased reabsorption of sulphate in the mother’s kidneys [24,25]. While sulphate is also a constituent of food, little is known about minimal requirements and average dietary intake levels of this nutrient for optimal growth and maintenance of health [26].

In the context of the role of the RSI in human development and the potential involvement of its biochemical mediators in the aetiology of DDH, an exploratory study into markers for oxidative stress and sulfation reactions was conceived. We hypothesised that DDH would be associated with increased oxidative stress and a reduced availability of sulphur compounds critical to sulfation reactions and cellular energy production.

## 2. Materials and Methods

**Figure 1:**
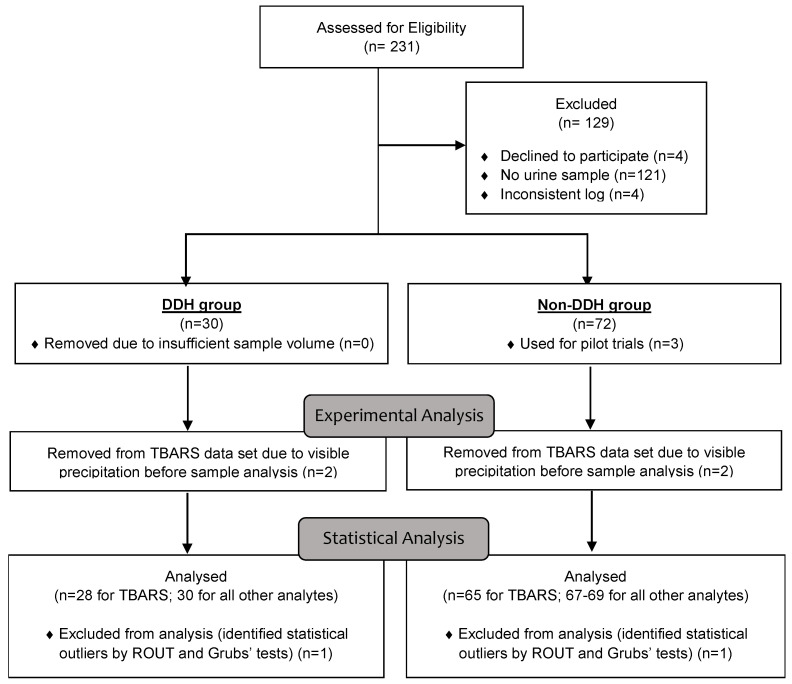
Flow chart demonstrating inclusion/exclusion criteria.

**Enrolment, sample collection, and storage:** Following full ethical approval (REC: 17/YH/0442), infants were consented from the routine hip screening clinic of a tertiary referral children’s hospital in England (Figure 1). Infants up to 12 weeks of age were included, whether diagnosed with DDH or screened and discharged with normal hips. Infants with medical co-morbidities were excluded. Spot urine samples were collected using cotton wool balls placed in the infants’ nappies whilst they awaited their appointment. The cotton wool was removed and compressed at the end of the visit. A minimum of 0.5 mL was required for sample analysis. Urine was considered a suitable biofluid for the measurement of metabolites, due to the relative ease with which it can be obtained from young infants in a non-invasive manner.

Demographic data were securely stored, including age at urine collection, medical history, and the presence or absence of DDH. All of the samples were anonymised at source, and those performing laboratory analyses remained blinded to all demographic and medical data. The samples were aliquoted into cryovials and frozen at −80 °C until analysis.

The hypothesis was explored by measuring concentrations of the stable end-products of reactive species/metabolites in infants with and without DDH. As renal function and fluid status are difficult to ascertain in young infants during routine clinical screening, creatinine and osmolality were quantified to enable adjustment of metabolite concentrations for urinary output and overall muscle mass.

**Thiosulphate, sulphate, nitrate, and phosphate:** Ion chromatography–mass spectrometry (IC-MS) was used to quantify the concentration of sulphate (SO_4_^2−^), nitrate (NO_3_^−^), thiosulphate (S_2_O_3_^2−^), and phosphate (PO_4_^3−^) in urine samples due to its high sensitivity and selectivity for these metabolites [27]. The samples were injected undiluted (neat) and diluted (1:100 with ultra-pure water) onto the IC-MS system (Dionex ICS-5000 with ISQ-EC detector, Thermo Fisher Scientific, Hemel Hempstead, UK) using an AS-AP autosampler. Sulphate, nitrate, thiosulphate, and phosphate were separated on a Dionex IonPac AS16 2 × 250 mm analytical column kept at a constant temperature of 30 °C. An eluent generator with a potassium hydroxide cartridge was used to produce the concentration gradient (a linear gradient from 20 mM to 30 mM between 0 and 4.0 min, after which it was held constant at 30 mM until 7.0 min, followed by a steep gradient up to 10 mM, which was kept for 2 min and reversal to 20 mM for equilibration until 12 min) at a constant flow rate of 0.38 mL/min and a suppressor current of 95 mA. The total run time was 12 min with retention times of 3.2, 3.9, 5.5, and 6.3 min for nitrate, sulphate, thiosulphate, and phosphate, respectively. The quadrupole detector was coupled to the IC system via an electrospray ionisation interface (ESI) operated in negative SIM mode at 62.1, 113, and 97.1 *m*/*z* for the detection of nitrate and the mono/di-protonated forms of thiosulphate, sulphate, and phosphate (HS_2_O_3_^−^, HSO_4_^−^, and H_2_PO_4_^−^), respectively. The capillary voltage was kept at 2.9 kV, whilst the vaporizer and ion transfer tube temperatures were 400 °C and 180 °C, respectively. The cone voltage was 20 V for all analytes. The sheath and auxiliary gas pressures were 50 psig and 2 psig, respectively. The concentration of anions in the urine sample was calculated by comparison of peak areas in the sample to peak areas in authentic standards of known concentrations. The sensitivity of the method is 0.1 µM for thiosulphate and nitrate and 0.5 µM for sulphate and phosphate.

*Nitrite***:** Nitrite (NO_2_^−^) levels in the urine samples were determined by high-performance liquid chromatography (HPLC) combined with a post-column diazo coupling reaction essentially as described [28]. The urine samples were injected onto the HPLC system (Eicom NOx Analyser ENO-20) using an autosampler (Gilson 234). The mobile phase was kept at a flow rate of 0.33 mL/min, while the reactor solution was being delivered to the reaction loop at a flow rate of 0.11 mL/min. Nitrite was separated from other anions on an analytical/separation column (NO-PAK, EiCom). Following separation, nitrite was eluted from the column and mixed with the reactor solution (Griess reagent) generating a pink/red diazo compound, the intensity of which was measured by absorbance at 540 nm in the detector cell. The concentration of nitrite in the urine sample was calculated by comparison to the nitrite peak area of aqueous standards of known concentrations. The total run time was 10 min with a retention time (RT) of 4.4 min for nitrite. The sensitivity is 0.1 µM at an injection volume of 20 µL.

*TBARS***:** TBARS (thiobarbituric acid reactive substances) is an assay for estimating lipid peroxidation products that has been used extensively as a marker of oxidative stress [29]. This colourimetric method is based on the reaction of malondialdehyde (MDA) and related reactive aldehydes generated from the ROS-induced breakdown of unsaturated lipids/fatty acids with thiobarbituric acid. 1,1,3,3,3-tetramethoxypropane was used to generate MDA with 0.6 N trichloroacetic acid for acidification and thiobarbituric acid as the colour reagent. The standard solution was made by adding 41 μL of 1,1,3,3,3-tetramethoxypropane into 500 mL MQ water (vortexed, protected from light for 30 min). The samples were prepared using the 1:1 ratio of 150 μL sample and TBARS reagent. These were then incubated at room temperature for 15 min, followed by centrifugation at ≥12,000× *g* for 4 min. One hundred and fifty μL of all standards and samples was added to cryovial with 75 μL of TBARS colour reagent. Sixty-seven μL of each was then transferred into half-well clear 96-well microplates after 2 h incubation at 80 °C. The MDA-TBA adduct (pink chromogen) formed from the reaction of MDA in samples with TBARS was measured, in duplicates, using a plate reader monitoring the absorbance at 532 nm.

*Creatinine***:** Biochemical markers are essential for providing information about kidney function. Creatinine originates from the spontaneous breakdown of creatine phosphate (in muscle) and is excreted by the kidneys at a constant rate. It is an easy and simple way of assessing kidney function and provides information about overall muscle mass. Creatinine was quantified using a commercial 96-well plate assay kit (MAK080; Sigma-Aldrich, Gillingham, UK), the principle of which is based on the Jaffe reaction, according to the standard protocol using a five-fold dilution of urine. The results were confirmed using a validated tandem mass spectrometry (LC-MS/MS) method [30]. While absolute values differed somewhat, data from both methods were closely associated (r = 0.918; *p* < 0.0001); the data depicted in the figures were from LC-MS/MS determinations.

*Osmolality***:** Osmolality is the optimum method for normalisation of urine metabolomics analyses [31,32]. To assess the infants’ hydration status, the concentration of dissolved solutes was determined in 20 µL aliquots of spot urines by measurement of freezing point depression using a Model 3320 micro-osmometer (Advanced Instruments, Wimborne, UK). Instrument performance was checked daily with reference solutions, and the arithmetric average of two independent observations was used for normalisation of analyte concentrations. In order to preserve the original analyte concentration units we calculated a normalisation factor by defining the median osmolality of the healthy infant group as 1 such that osmolality values below and above this resulted in factors smaller and greater than 1, respectively.

**Statistical Analysis:** Statistical analysis was performed using GraphPad Prism 9.2.0 (San Diego, CA, USA). The normality of distributions was tested using Anderson-Darling and Kolmogorov–Smirnov tests; since most parameters were found to be non-normally distributed, this assumption was considered to apply for all analytes. Median and interquartile ranges were used to represent non-parametric data. Statistical significance was established using Mann–Whitney tests (non-parametric) at *p* < 0.05. Owing to its novel nature, the effect size of the study is unknown, and thus no power calculations could be applied.

## 3. Results

Of 231 eligible infants, consent for urine collection was received from 227 families, of which 102 infants produced urine for collection. Small numbers were excluded due to the urine volume being insufficient, for use in pilot tests, due to urine contamination, and due to statistical anomalies determined on ROUT and Grubs’ test (Figure 1). This left samples from 30 infants with sonographically confirmed DDH and from 69 infants with normal hips for laboratory analysis. Ages ranged from 13–61 days, with both cohorts evenly matched for age. The DDH cohort contained a significantly higher proportion of females, in keeping with the known sex association with DDH (Table 1).

**Figure 2:**
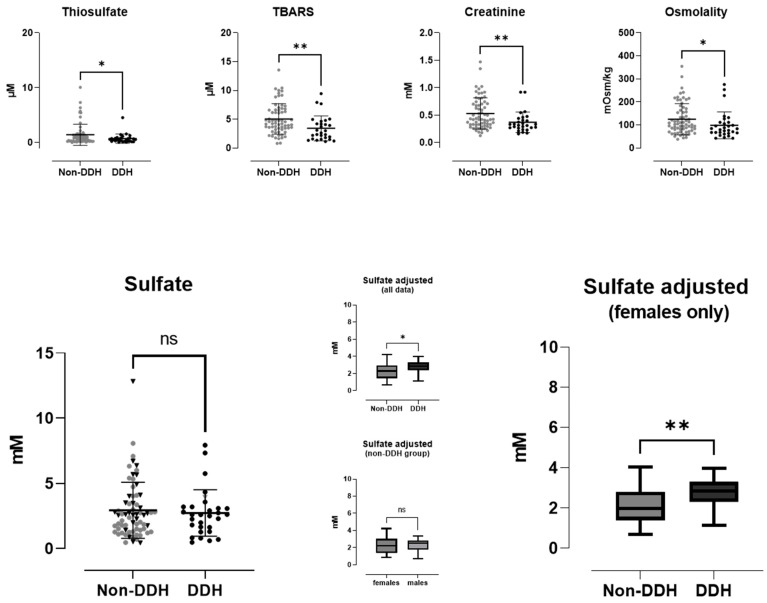
**Upper row:** Urinary concentrations of thiosulphate, thiobarbituric acid reactive substances (TBARS), creatinine, and osmolality in infants with DDH compared to age-matched controls (non-DDH). **Lower Row:** *Left*: Distribution of unadjusted urinary sulphate concentrations in non-DDH and DDH infants (triangles—boys; circles—girls). *Middle*: After adjustment for osmolality, urinary sulphate concentrations differed between DDH and non-DDH babies whereas in the non-DDH group, sulphate concentrations showed no difference between males and females. *Right:* Differences between groups are even more marked if only girls were compared. Scatter plots show non-normalised data with significant differences in analyte concentrations between the non-DDH and DDH group (distribution of other analytes with non-significant (ns) differences between groups not shown) whereas the box and whisker plot for sulphate depicts osmolality-adjusted concentrations. * **= *p* < 0.05**. ** **= *p* < 0.005**.

Significant differences in concentrations were found for thiosulphate, TBARS, and creatinine between the DDH and non-DDH groups (Table 1), with a considerable spread of values (Figure 2). In contrast to earlier reports [12] and our hypothesis that DDH would be associated with elevated oxidative stress, thiosulphate concentrations and the oxidative stress marker, TBARS, were significantly lower in the DDH group. Since urinary creatinine concentrations were also lower in the DDH group (median: 0.425 vs. 0.314 mM, *p* = 0.0048), anion concentrations could not be normalised across the entire cohort for renal function using creatinine. A determination of osmolality revealed that not only creatinine but indeed the concentration of all solutes was lower in DDH urines (103 vs. 82 mOsm/kg in non-DDH vs. DDH; *p* = 0.034). Since it is unlikely that the majority of non-DDH infants were dehydrated, these findings indicate that DDH and non-DDH infants differ in hydration status and/or urinary output, with the former producing more diluted urine. When urinary creatinine levels were adjusted for osmolality, differences between groups disappeared (438 vs. 428 µM, *p* = 0.929). Thus, creatinine was not used for normalisation.

When the concentrations of other analytes were adjusted for osmolality, a different pattern of similarities and differences became apparent. Following normalisation for hydration status/urinary output, now only sulphate was significantly different between the groups, with higher urine concentrations in DDH infants compared to the controls (Figure 2); all other analyte levels were similar between the groups. Since sex was unevenly distributed between the non-DDH and DDH groups (see Table 1), a concern arose that the observed differences might be a reflection of sexual dimorphism. With only a single male in the DDH group, a subgroup analysis would have been meaningless. Urinary sulphate levels in the control group tended to be higher in male than in female infants, but this difference was not significant. When all males were removed from either group, the difference between the DDH and non-DDH groups became statistically even more significant (see Figure 2), corroborating our main findings and dispelling concerns that sex differences could have confounded this outcome.

## 4. Discussion

This novel study on potential biochemical mechanisms contributing to the aetiology of DDH has shown, for the first time, significant differences in inorganic metabolites linked to nutrition, stress sensing, and glycosaminoglycan biology in infants with DDH. The potential implications of this new information are considerable, for both screening and treatment strategies. In the UK, 1 in 200 babies are diagnosed with DDH [1], yet the screening programme is far from optimal [33]. If DDH could be linked to specific metabolic markers, efficiencies would be instantaneous for the national screening programme. Current treatment for DDH involves a harness for the first few months of life or surgery if detected later. Neither is perfect. The findings of this study can open the door to exploration of a biochemical treatment for DDH. Due to the hip developmental origins in the first trimester [24,25,26], this could be as simple as maternal nutritional supplementation, as seen with folic acid in pregnancy for spina bifida, and/or dietary counseling of pregnant women.

Fitting to its novel nature, these results cannot be comprehensively compared to other studies as there is little to no literature available on these analytes in urine samples for this age group. A targeted metabolomic approach was chosen to quantify the urinary concentrations of metabolites using a combination of selective and sensitive analytical methods. Unexpectedly, osmolality was significantly lower in DDH versus non-DDH, indicating a more diluted urine in infants with DDH. We cannot explain this finding, yet its method of determination is unambiguous since it uses a physical property that takes into account all dissolved substances irrespective of structure, charge, and size. Further to the significant differences in urinary concentrations of thiosulphate, TBARS, and creatinine in infants with DDH, when the original values for the metabolites were adjusted for osmolality (i.e., urine dilution) only one species, sulphate, emerged to be different between groups. The lack of information on the circulating concentrations of sulphate in blood makes it difficult to assess whether alterations in excretion and/or reabsorption account for these differences and prevents the drawing of definitive conclusions from this study alone. Nevertheless, considering the importance of sulphate for skeletal development as well as its precursor (H_2_S) for redox signalling and physiology, this result represents an important new finding that promises to inform future research directions.

Contrary to our hypothesis, urinary sulphate levels were higher in babies with DDH compared to non-DDH controls. Higher sulphate concentrations in urine may be indicative of enhanced excretion of excess systemic sulphate due to either higher oral intake, greater endogenous production, or both. Of note, sulphate concentrations in human milk have been reported to be 13-fold lower than in formula milk [34]. Increased urinary excretion of sulphate has been associated with increased protein consumption (from fortified breast milk or formula) and associated methionine overload [35]. Dietary information was not collected in this study but must be a focus of future analyses. Greater availability of sulphate may translate into enhanced sulfation and altered structure of chondroitin/heparan and other glycosaminoglycans, as reported for idiopathic pulmonary fibrosis [36]. Alternatively, greater urinary sulphate levels may be the result of lower renal reabsorption of sulphate (sulphate wasting) and be associated with similar or lower levels of circulating sulphate secondary to enhanced renal excretion, e.g., from aberrant sulphate transporter expression/activity in the proximal tubules. Our current understanding of the handling of sulphate by the human body, which involves endogenous formation, metabolism by the gut as well as reabsorption and secretion in the kidneys, is incomplete. Even less is known about foetal sulphate requirements/demands for growth and development. What is becoming increasingly clear, however, is that the placental sulphate supply from mother to foetus is governed by complex adaptive processes that extend well beyond dietary intake, including environmental factors, over-the-counter drugs that are metabolised by conjugation to sulphate such as acetaminophen (paracetamol), and psychosocial stress [37]. These stressors may not only affect maternal endocrine function and stress signalling but also the nutritional supply of the foetus with sulphate or its precursor cysteine. Whether any of these processes are involved in joint deformation in DDH remains to be investigated.

Our preliminary results found significantly lower creatinine concentrations in the DDH group than in the control group. This could have represented impaired renal function, low protein intake, or reduced muscle mass. If true, this would have been a significant finding, hence the group’s efforts to independently confirm creatinine differences using an orthogonal method of quantification (see Methods section). However, after normalisation of urinary creatinine for osmolality, that difference disappeared. Thus, there is no evidence for any major difference in renal function, lean body mass (muscle), or protein load in DDH. Similar to the considerations discussed for sulphate and other analytes, this demonstrates the importance of an appropriate control for hydration status (and thus urine dilution) when using spot urine samples for biochemical analyses, particularly in young children.

The collected specimens were spot urines obtained at different times of the day and year; thus, we cannot exclude that chronobiological differences in production and/or excretion of the biomarkers measured may have influenced the results. No information on the nutritional status of the children, nor their mothers, was collected, including which infants were breast- or formula-fed. This information is important in further work, including a full maternal dietary and vitamin supplementation history, due to the potential dietary influence on sulphate-related pathways. Furthermore, whilst the groups were well matched for age, there was a sex discrepancy within the DDH group consisting of more females than males, in keeping with DDH being known to affect girls more than boys. Further studies must account for sex matching, lest there are unknown differences in urinary sulphate between male and female infants.

## 5. Conclusions

DDH is one of the most common and challenging congenital skeletal disorders with an immensely unclear aetiology. To the best of our knowledge, this is the first study to investigate the potential role of specific anions in the aetiology of DDH. The significant differences between infants with DDH and age-matched infants without DDH point to a potential problem in sulphur metabolism in DDH that may translate into alterations in skeletal development. This has enormous implications for new avenues of screening for DDH or even prevention by dietary supplementation.

## Figures and Tables

**Table 1:** Infant age and urine analyte concentrations. Age data (normal distribution) is shown as mean ± SD. Concentrations are given as median (interquartile range) as the data were log-normally distributed (non-parametric).

	Non-DDH (*n* = 69)	DDH (*n* = 30)	*p* Value
Age (days)	41.5 ± 9.3	39.8 ± 12.5	0.478
Sex (F:M)	43:26	29:1	0.0002 **
Thiosulphate (μM)	0.879 (1.458–0.292)	0.573 (0.802–0.245)	0.041 *
Sulphate (mM)	2.566 (3.504–1.423)	2.606 (3.171–1.643)	0.925
Nitrite (μM)	0.610 (1.425–0.390)	0.755 (0.953–0.465)	0.983
Nitrate (mM)	0.401 (0.740–0.293)	0.423 (0.621–0.238)	0.487
Phosphate (mM)	2.483 (5.748–0.798)	3.825 (6.012–0.660)	0.931
Creatinine (mM)	0.425 (0.705–0.328)	0.324 (0.417–0.269)	0.0048 **
Osmolality(mOsm/kg)	103 (78.5–161)	82.0 (66.5–111)	0.034 *
TBARS (μM)	*Non-DDH* (*n = 67*) 4.320 (3.440–6.610)	*DDH* (*n = 26*) 2.820 (1.755–4.323)	0.004 **

*, ** Shows statistical significance at *p* < 0.05 or *p* < 0.005, respectively.

## Data Availability

The data that support the results in this study are available from the corresponding authors upon reasonable request.
